# Is being a regular player with fewer teammates associated with musculoskeletal pain in youth team sports? A cross-sectional study

**DOI:** 10.1186/s12891-017-1470-z

**Published:** 2017-03-14

**Authors:** Takafumi Abe, Masamitsu Kamada, Jun Kitayuguchi, Shinpei Okada, Yoshiteru Mutoh, Yuji Uchio

**Affiliations:** 1 Center for Community-Based Health Research and Education (CoHRE), Organization for the Promotion of Project Research, Shimane University, 223-8 Enya-cho, Izumo, Shimane 693-8501 Japan; 2 Physical Education and Medicine Research Center UNNAN, 328 Uji, Kamo-cho, Unnan City, Shimane 699-1105 Japan; 3 Department of Orthopaedics, Shimane University School of Medicine, 89-1 Enya-cho, Izumo City, Shimane 693-8501 Japan; 4 Department of Social and Behavioral Sciences, Harvard T.H. Chan School of Public Health, 401 Park Drive, 4th floor, Boston, MA 02215 USA; 5 Department of Health Promotion and Exercise, National Institute of Health and Nutrition, 1-23-1 Toyama, Shinjuku-ku, Tokyo 162-8636 Japan; 6 Japan Society for the Promotion of Science, 5-3-1, Kojimachi, Chiyoda-ku, Tokyo, 102-0083 Japan; 7 Physical Education and Medicine Research Foundation, 6-1 Nunoshita, Tomi City, Nagano 389-0402 Japan; 8 Department of Preventive Medicine and Public Health, Tokyo Medical University, 6-1-1, Shinjuku, Shinjuku-ku Tokyo, 160-8402 Japan; 9 The Research Institute of Nippon Sport Science University, 7-1-1 Fukasawa, Setagaya-ku Tokyo, 158-0081 Japan

**Keywords:** Sports, Social environment, Musculoskeletal disease, Substitute, Adolescent, Epidemiology

## Abstract

**Background:**

Musculoskeletal pain (MSP) is a commonly reported symptom in youth sports players. Some sports-related risk factors have been reported, but previous studies on extrinsic risk factors did not focus on management of team members (e.g., regular or non-regular players, number of players) for reducing sports-related MSP. This study aimed to examine the association of playing status (regular or non-regular players) and team status (fewer or more teammates) with MSP in youth team sports.

**Methods:**

A total of 632 team sports players (age: 12–18 years) in public schools in Unnan, Japan completed a self-administered questionnaire to determine MSP (overall, upper limbs, lower back, and lower limbs) and playing status (regular or non-regular players). Team status was calculated as follows: teammate quantity index (TQI) = [number of teammates in their grade]/[required number of players for the sport]. Associations between the prevalence of pain and joint categories of playing and team status were examined by multivariable-adjusted Poisson regression.

**Results:**

A total of 272 (44.3%) participants had MSP at least several times a week in at least one part of the body. When divided by playing or team status, 140 (47.0%) regular and 130 (41.7%) non-regular players had MSP, whereas 142 (47.0%) players with fewer teammates (lower TQI) and 127 (41.8%) players with more teammates (higher TQI) had MSP. When analyzed jointly, regular players with fewer teammates had a higher prevalence of lower back pain compared with non-regular players with more teammates (21.3% vs 8.3%; prevalence ratio = 2.08 [95% confidence interval 1.07–4.02]). The prevalence of MSP was highest in regular players with fewer teammates for all other pain outcomes, but this was not significant.

**Conclusion:**

Regular players with fewer teammates have a higher risk of lower back pain. Future longitudinal investigations are required.

**Electronic supplementary material:**

The online version of this article (doi:10.1186/s12891-017-1470-z) contains supplementary material, which is available to authorized users.

## Background

The prevalence rate of musculoskeletal pain (MSP) in adolescents in the general population ranges from 4% to 40% [1]. In school-aged youth, the point prevalence of lower back pain is 10.2% and the lifetime prevalence is 28.8% [2]. MSP in youth is associated with functional disability [3], lower quality of life [4], and future risk of MSP for adulthood [5]. Therefore, prevention of MSP in adolescents is important for health throughout the life span.

Participation in sports among adolescents has physical, psychological, and social health benefits [6, 7]. However, one of the negative effects of participation in organized sports is an increased risk of musculoskeletal problems. Previous reviews have shown that extrinsic risk factors of sports-related MSP and injuries include the type of sport, competitive level, intensity of physical training, acute spinal trauma, over-training, insufficient recovery between activities, and greater exposure to injury [8–13]. Furthermore, a recent longitudinal study showed that an increase in time in playing organized sports is linearly associated with a risk of MSP in adolescents [14].

Management of members in team sports is important for reducing physical loading and recovering from fatigue for physical performance [15]. Orchard [16] suggested that more research for substitute and interchange rules in competitive team sports is needed to reduce fatigue for preventing injury. However, previous studies on extrinsic risk factors did not focus on management of team members (e.g., regular or non-regular players, number of players) for reducing sports-related MSP. Regular players with limited numbers of teammates have a heavier physical burden because of a longer duration of playing time compared with non-regular players with sufficient numbers of teammates. To the best of our knowledge, no studies have examined the association between playing status (regular or non-regular players) and team status (number of teammates) with MSP in youth team sports.

Therefore, this study aimed to investigate the associations of playing status (regular or non-regular players) and team status (fewer or more teammates) with MSP (overall, upper limbs, lower back, and lower limbs) in youth team sports.

## Methods

### Study design

The present cross-sectional study was carried out concomitantly with a previous study [14]. We hypothesized that regular players with fewer teammates relative to the required number of players in team sports have a higher probability of having pain compared with non-regular players with more teammates. This study protocol was previously approved by the research ethics committee of the Physical Education and Medicine Research Center UNNAN (H19-7-23-5). The study procedure, analysis, and description were reported according to the Strengthening the Reporting of Observational Studies in Epidemiology statement [17].

### Participants

**Fig. 1:**
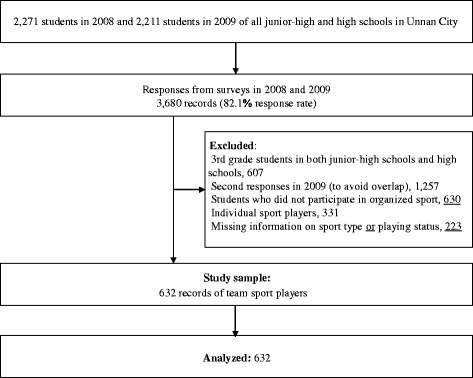
Flowchart of the study

All junior high schools (seven schools; seventh to ninth grades; students’ age: 12–15 years) and high schools (three schools; 10th–12th grades; students’ age: 15–18 years) in Unnan City (population: 43,520; area: 553.4 km^2^), Shimane, Japan, participated in the previous study [14]. In October 2008 and 2009, self-administered questionnaires were distributed to all students (2,271 students in 2008 and 2,211 students in 2009) and returned through schools. Students who received special needs education and those with invalid (blank) responses were excluded. The total response rate was 82.1% (3,680 responses from two surveys in 2008 and 2009). Third (final) grade students in junior high schools and high schools in 2008 were excluded from the study. These students were excluded because they had stopped their organized sports activity (if they participated) since July to the end of the academic year (March) to focus on academic work, in accordance with Japanese school custom. Additionally, students pursuing individual sports (*n* = 331), those pursuing no sports (*n* = 630), and those with invalid responses (missing data) on the type of sport or playing status (*n* = 223) were excluded. We used only the first-year data of each individual (i.e., first and second grades in 2008 and first grades in 2009) to avoid overlap. As a result, 632 eligible responses were analyzed for the present study (Fig. 1). An explanation of the students’ right to decline to answer any questions and general information about the study were provided in a letter to parents or guardians of the students who answered the questionnaire. Students signed their name on the questionnaire to give their assent to participate in the study and returned the completed questionnaire in an anonymous envelope. Individual answers were not shared with teachers or sports coaches.

### Data collection

Self-administered questionnaires included questions on sex, age, weight and height (used for calculating body mass index [BMI] in kg/m^2^), organized sports activity (h/wk from hours on weekdays and the weekend), sleep time (h/d from time in and out of bed), and screen time (h/wk of TV viewing and game playing time on weekdays and the weekend). For students who participated in organized sports activity, questions regarding the nature of the sport in which they were registered (e.g., baseball, basketball) and whether they were regular players (Do you currently participate in competitions as a regular player or represent your team? [yes/no]) were also asked. In this study, organized sports activity was defined as sports activities that occurred after school programs on weekdays and/or weekends or activities that were organized by sports clubs.

### Teammate quantity index

To estimate the opportunity to actively participate in organized sports competitions and games and the possible physical burden among regular players with fewer teammates, a teammate quantity index (TQI) was calculated as follows: TQI = [number of teammates in their grade]/[required number of players for the sport]. The registration information regarding the number of teammates in sports clubs was reported by the schools that managed the clubs. The required numbers of players for each sport were assigned as follows: baseball and softball = 9; basketball = 5; soccer = 11; and volleyball = 6. For example, in the situation of a baseball team with 14 students in the second grade, the TQI for these second grade students is 14 divided by 9 (i.e., TQI = 1.6). A higher TQI indicates more teammates in the same grade in their team with consideration for each sport. TQI was dichotomized as high or low, using the median value (1.3) as the cutoff.

### Musculoskeletal pain

MSP was assessed using a questionnaire [see Additional file 1]. Details of the questionnaire are described elsewhere [14]. Briefly, pain was defined by the frequency and the part of the body area that was painful. Pain should be present at least once a week in at least one part of the body. The test-retest reliability over 7 days of the pain questionnaire was tested and found to be acceptable (Cohen’s kappa for pain in any location = 0.67). The agreement test (criterion validity test) with a face-to-face interview by health professionals (e.g., public health nurse, health fitness programmer) who were trained by an orthopedist also showed moderate agreement (Cohen’s kappa = 0.52).

### Statistical methods

We compared the prevalence of pain in students with different playing status (regular or non-regular player) and team status (lower TQI or higher TQI), using a chi-squared test.

Our hypothesis was that regular players with fewer teammates relative to the required number of players in the relevant sport have a higher probability of having pain, compared with non-regular players with more teammates. First, we evaluated whether playing status (regular or non-regular player) or team status (number of teammates) was associated with the prevalence of pain separately, using multivariable-adjusted Poisson regression [18]. Second, we tested the interaction between playing status (regular or non-regular players) and the TQI for prevalence of pain. Additionally, we defined the joint categories of playing status and team status as 1) non-regular players with a high TQI, 2) non-regular players with a low TQI, 3) regular players with a high TQI, and 4) regular players with a low TQI. We examined the association of these categories with the prevalence of pain, using Poisson regression. Location-specific (upper limb, lower back, and lower limb) pain analyses were also performed. Poisson regression was used to calculate prevalence ratios (PRs), which are more easily interpreted than odds ratios [19]. Adjustments were made by sex, age, BMI, sleep length, screen time, and school. BMI, sleep length, and screen time were included in the model as categorical variables divided by tertiles within each grade with the lowest groups as references. School was included in the model as a fixed effect rather than a random effect. The fixed effect was used because all school (cluster) level confounders were able to be adjusted, regardless of whether they were measured [20]. School was considered to be more appropriate than school class considering the scarce distribution of outcomes in each class. Additionally, students within the same schools shared some potential confounders of pain beyond classes and grades (e.g., sports clubs, physical education teachers, sports facilities, and environment).

Missing information was processed using multiple imputation (n datasets = 10) under the missing at random assumption [21, 22]. Each imputation was based on regression models including variables used in the main regression analyses. The 10 imputed datasets were analyzed independently and combined for inference, accounting for variability of imputation [21]. For sensitivity analyses, the analyses were repeated using only cases with no missing values (*n* = 541). Analyses (two-sided α < 0.05) were carried out using SAS version 9.3 (SAS Institute Inc., Cary, NC).

## Results

### Participants

Table 1 shows the participants’ characteristics with and without pain before multiple imputations. Of the 632 students in the analysis, 40.7% were girls, and students had a mean (standard deviation: SD) age of 13.8 (1.5) years. The mean (SD) time spent in organized sports activity was 18.4 (6.0) h/wk among the analyzed students (team sports players), which was higher than that of excluded students (individual sports: 15.7 h/wk; invalid response: 16.3 h/wk). The TQI ranged from 0.1 to 2.8 and had a mean (SD) of 1.3 (0.6) among team sports students. The proportion of regular players was 48.4%.

**Table 1:** Characteristics of the study participants and non-participants

	Analyzed participants				Excluded students			
	Total	No pain	Pain	*p* value^c^	Individual sports^d^	No sports	Invalid response^e^	*p* value^f^
Number of participants, n (%)	632	342	272 (44.3)		331	630	223	
Sex, female, %	40.7	40.6	40.8	0.96	40.5	78.4	34.1	0.019
Age, years	13.8 ± 1.5	13.7 ± 1.5	14.0 ± 1.6	<0.05	14.7 ± 1.6	14.4 ± 1.6	13.5 ± 1.4	<0.001
Body mass index, kg/m^2^	19.0 ± 2.4	18.9 ± 2.5	19.1 ± 2.3	0.21	19.9 ± 3.2	19.6 ± 2.8	18.7 ± 2.6	<0.001
Sleep length, h/d	7.8 ± 1.8	7.9 ± 2.1	7.6 ± 0.9	<0.05	7.3 ± 0.9	7.2 ± 1.0	7.8 ± 0.9	<0.001
Screen time, h/wk	20.7 ± 10.9	21.5 ± 11.1	19.6 ± 10.6	0.06	21.5 ± 10.7	20.6 ± 12.5	21.5 ± 10.7	0.185
Sports activity time, h/wk	18.4 ± 6.0	17.7 ± 5.6	19.1 ± 6.3	<0.01	15.7 ± 6.1	-	16.3 ± 10.7	<0.001
Regular player, %	48.4	46.5	51.9	0.19	60.2	-	45.2	0.266
Type of team sport, n (%)^a^								
Baseball	176	100	73 (42.2)		-	-	-	
Softball	102	56	42 (42.9)		-	-	-	
Basketball	111	50	57 (53.3)		-	-	-	
Soccer	40	21	17 (44.7)		-	-	-	
Volleyball	200	113	82 (42.1)		-	-	-	
Other	3	2	1 (33.4)		-	-	-	
Teammate quantity index^b^	1.3 ± 0.6	1.3 ± 0.5	1.4 ± 0.6	0.16	-	-	-	

Values are numbers and percentages for categorical variables and mean ± standard deviation for continuous variables. Sample sizes of number counts vary because of missing values

^a^Prevalence of pain (%) within the type of sport

^b^Teammate quantity index = [number of teammates in their grade]/[required number of players for the sport]

^c^Comparison between students with and without pain by t-test or Pearson’s chi-squared test

^d^Track and field, Soft tennis, Table tennis, Badminton, Kendo, Judo, Karate and Swimming

^e^Missing information on sport type and playing status

^f^Comparison of included and excluded groups by chi-squared test or Kruskal-Wallis test

### Prevalence of musculoskeletal pain

A total of 272 (44.3%) players had MSP in any location. The most prevalent location of pain was the lower limbs (181 cases, 29.4%), followed by the upper limbs (90 cases, 14.5%) and lower back (77 cases, 12.4%). When grouped according to playing status or team status, 140 (47.0%) regular and 130 (41.7%) non-regular players had MSP (*p* = 0.19), whereas 142 (47.0%) players with fewer teammates (lower TQI) and 127 (41.8%) players with more teammates (higher TQI) had MSP (*p* = 0.19).

### Associations with musculoskeletal pain

Table 2 shows the association of regular status and the TQI with the prevalence of pain among team sports players. In the multivariable analysis, regular players with a low TQI (i.e., fewer teammates) had a higher prevalence of lower back pain compared with non-regular players with a high TQI (PR = 2.08 [95% CI 1.07–4.02]). Although the associations with pain overall and other locations were not significant, the prevalence of MSP was highest in regular players with a low TQI for all pain outcomes. The interaction between regular status and the TQI was not significant for all pain outcomes (*p* ≥ 0.13). However, sensitivity analyses by using the complete case data indicated a similar association and significant interaction (*p* = 0.02) for lower back pain to primary analyses.

**Table 2:** Associations of regular status and the teammate quantity index with the prevalence of pain

	Non-regular players with a high TQI^a^	Non-regular players with a low TQI^a^	Regular players with a high TQI^a^	Regular players with a low TQI^a^	p for interaction
Pain					
Overall, case/n (%)	80/189 (42.3)	48/121 (39.7)	47/116 (40.5)	93/179 (52.0)	
Crude PR (95% CI)	1 (reference)	0.94 (0.66–1.35)	0.95 (0.67–1.33)	1.22 (0.91–1.64)	0.06
Adjusted PR (95% CI)^b^	1 (reference)	0.94 (0.64–1.37)	0.98 (0.69–1.41)	1.24 (0.89–1.74)	0.13
Specific location					
Upper limb, case/n (%)	23/189 (12.2)	15/126 (11.9)	16/117 (13.7)	35/179 (19.6)	
Crude PR (95% CI)	1 (reference)	0.97 (0.51–1.83)	1.10 (0.59–2.05)	1.59 (0.96–2.64)	0.32
Adjusted PR (95% CI)^b^	1 (reference)	0.69 (0.34–1.39)	1.11 (0.57–2.16)	1.04 (0.57–1.89)	0.41
Lower back, case/n (%)	16/192 (8.3)	8/123 (6.5)	13/116 (11.2)	39/183 (21.3)	
Crude PR (95% CI)	1 (reference)	0.90 (0.39–2.08)	1.28 (0.63–2.61)	2.45 (1.38–4.36)	0.11
Adjusted PR (95% CI)^b^	1 (reference)	0.92 (0.38–2.22)	1.10 (0.52–2.33)	2.08 (1.07–4.02)	0.19
Lower limb, case/n (%)	53/189 (28.0)	36/123 (29.3)	33/115 (28.7)	55/179 (30.7)	
Crude PR (95% CI)	1 (reference)	1.04 (0.69–1.56)	1.00 (0.66–1.51)	1.13 (0.78–1.62)	0.13
Adjusted PR (95% CI)^b^	1 (reference)	1.06 (0.69–1.62)	1.03 (0.67–1.58)	1.19 (0.79–1.80)	0.27

*Abbreviations*: *CI* confidence interval, *PR* prevalence ratio, *TQI* teammate quantity index. Sample sizes (denominators) of number counts vary because of missing values, although the PRs were calculated by multiple imputed data sets. Students were divided into two categories by the median of the index (<1.3, 1.3+). A high TQI indicates more teammates in their clubs with consideration for each sport type.

^a^TQI = [number of teammates in their grade]/[required number of players for the sport]

^b^Adjusted for sex, age, body mass index, sleep length, screen time, school, and sports activity time

When playing status (regular or non-regular player) and team status (number of teammates) were analyzed separately, regular status alone was significantly associated with lower back pain (PR = 1.95 [95% CI 1.19–3.18]). However, regular status alone was not significantly associated with pain overall, and upper limb and lower limb pain (*p* ≥ 0.1). Similarly, the TQI itself showed no significant linear relationship with pain overall (*p* = 0.31) or pain in any of the three locations (*p* ≥ 0.32).

## Discussion

The present study examined the association of playing status (regular or non-regular players) and team status (fewer or more teammates) with MSP (overall, upper limbs, lower back, and lower limbs) in youth team sports. Regular players with fewer teammates had a two times higher prevalence of lower back pain compared with non-regular players with more teammates. To the best of our knowledge, the present study is the first to describe the association of playing status and team status with MSP. Adding to studies that show risk factors for sports-related MSP and injuries [8–13], our study found a novel joint association of regular-player status and fewer teammates with lower back pain.

There are several possible mechanisms underlying the association of regular players and fewer teammates with MSP. Regular players are considered to have a longer playing time and heavier physical burden in sports competitions and team training compared with non-regular players. A recent study reported a linear association between time in playing organized sports and the risk of MSP in adolescents [14]. In another study, the number of competitions per 100 days in team sports was positively associated with sports injuries [23]. Regular players are considered to play for a longer duration of time and thus they may have a higher risk of MSP. Additionally, rotation and interchange of teammates in team sports competitions and games are important for reducing the physical burden [16]. Teams with fewer members may not be able to rotate players frequently compared with teams with more members. Therefore, regular players with fewer teammates may not rest enough for a full recovery and have a higher risk of accumulating fatigue in the musculoskeletal system [24]. This situation then leads to a higher risk of MSP.

A recent study reported that the number of adolescents who play organized sports has decreased in several countries [25]. The participation rates of South Australians aged 10–13 years in one or more school or club sports decreased from 1985 to 1997/1999 [26]. In this previous study, boys’ participation in at least one sport declined from 87% to 76%, while among girls, participation fell from 80% to 71%. In Japanese children [27], the rate of participation in organized sports similarly decreased from 100% in 2002 to 79.4% in 2014, causing a decrease in the average number of members in sports clubs (26.7 members per club in 2002 and 21.8 in 2014). Howie et al. [28] reported that one-third of girls and boys after the age of 8 years in Western Australia dropped out of playing sports. For example, lower household income, main language spoken at home (non-English), lower parental education and child not taken to a sporting event were predictors of dropout from organized sports [29]. In addition, adolescent girls’ reasons for dropout may be loss of interest, lack of competence, insufficient time, body image, teasing, and feeling that sports are not a feminine activity [30]. Considering the results of the present study, a decrease in the number of youth sports players may further contribute to increased physical burden, and thus to an increase in risk of MSP, among the remaining players in the team. Preventive actions focused on youth sports players with small numbers of teammates may be necessary and will require multidisciplinary cooperation of stakeholders such as coaches, parents, and sports policy makers.

Students with MSP were older and had shorter sleep time and longer sports time than students without MSP. These findings are consistent with several previous reports in adolescents [8, 14, 31, 32].

This study has several strengths. First, this study recruited participants from all schools in the city. The response rate was relatively high, and therefore, the risk of selection bias was low. Additionally, our findings may have higher generalizability compared with studies that focused on limited elite athletes or injured patients. Second, MSP was measured by a reliable and valid questionnaire that was developed for this survey [14]. However, there are also several potential limitations in this study. First, the present study was a cross-sectional design and could not explain the causal relationship among playing and team status with MSP. Second, location-specific analysis had a limited sample size. The low statistical power could have led to underestimation of any associations. Third, although school registration information was used for team status (number of teammates), information on playing status (regular or non-regular players) was self-reported and may have been subject to recall bias. Other covariables were also self-reported, and their validity or bias is unknown. Finally, effects of unmeasured factors that may influence the sports-related MSP relationship, including puberty, smoking, mood, headache, tiredness, psychological factors, socio-economic status, cultural background and a history of participation in sports (e.g., length in years of participation), could not be accounted for in this study. For example, depression is known to be associated with higher pain prevalence [33] and lower sports participation [34]. Therefore, unmeasured confounding by depression might attenuate the positive association between regular-player status and MSP. Future longitudinal studies should be designed to investigate the associations of playing and team status with MSP.

## Conclusion

This cross-sectional study of adolescents playing team sports shows that regular players with fewer teammates have a two times higher prevalence of lower back pain compared with non-regular players with more teammates. Although there are no significant associations of MSP with pain overall and other locations, the prevalence of MSP is highest in regular players with fewer teammates for all pain outcomes. Individual physical burden may be higher in players actively participating in sports with fewer teammates. Future longitudinal studies are required to investigate the association of playing and team status with MSP.

## Additional file

Additional file 1: eQuestionnaire 1. Pain questionnaire for adolescents. (PDF 480 kb)

## Abbreviations

C: Confidence interval
MSP: Musculoskeletal pain
PR: Prevalence ratio
TQ: Teammate quantity index

## References

[1] King S, Chambers CT, Huguet A, MacNevin RC, McGrath PJ, Parker L, MacDonald AJ (2011). The epidemiology of chronic pain in children and adolescents revisited: a systematic review. Pain.

[2] Sato T, Ito T, Hirano T, Morita O, Kikuchi R, Endo N, Tanabe N (2008). Low back pain in childhood and adolescence: a cross-sectional study in Niigata City. Eur Spine J.

[3] Guite JW, Logan DE, Sherry DD, Rose JB (2007). Adolescent self-perception: associations with chronic musculoskeletal pain and functional disability. J Pain.

[4] Balague F, Ferrer M, Rajmil L, Pont Acuna A, Pellise F, Cedraschi C (2012). Assessing the association between low back pain, quality of life, and life events as reported by schoolchildren in a population-based study. Eur J Pediatr.

[5] Harreby M, Neergaard K, Hesselsoe G, Kjer J (1995). Are radiologic changes in the thoracic and lumbar spine of adolescents risk factors for low back pain in adults? A 25-year prospective cohort study of 640 school children. Spine (Phila Pa 1976).

[6] Eime RM, Young JA, Harvey JT, Charity MJ, Payne WR (2013). A systematic review of the psychological and social benefits of participation in sport for adults: informing development of a conceptual model of health through sport. Int J Behav Nutr Phys Act.

[7] Janssen I, Leblanc AG (2010). Systematic review of the health benefits of physical activity and fitness in school-aged children and youth. Int J Behav Nutr Phys Act.

[8] Balague F, Troussier B, Salminen JJ (1999). Non-specific low back pain in children and adolescents: risk factors. Eur Spine J.

[9] Jones GT, Macfarlane GJ (2005). Epidemiology of low back pain in children and adolescents. Arch Dis Child.

[10] DiFiori JP, Benjamin HJ, Brenner JS, Gregory A, Jayanthi N, Landry GL, Luke A (2014). Overuse injuries and burnout in youth sports: a position statement from the American Medical Society for Sports Medicine. Br J Sports Med.

[11] Valovich McLeod TC, Decoster LC, Loud KJ, Micheli LJ, Parker JT, Sandrey MA, White C (2011). National Athletic Trainers’ Association position statement: prevention of pediatric overuse injuries. J Athl Train.

[12] Emery CA (2003). Risk factors for injury in child and adolescent sport: a systematic review of the literature. Clin J Sport Med.

[13] McGuine T (2006). Sports injuries in high school athletes: a review of injury-risk and injury-prevention research. Clin J Sport Med.

[14] Kamada M, Abe T, Kitayuguchi J, Imamura F, Lee IM, Kadowaki M, Sawada SS, Miyachi M, Matsui Y, Uchio Y (2016). Dose-response relationship between sports activity and musculoskeletal pain in adolescents. Pain.

[15] Ronglan LT, Raastad T, Borgesen A (2006). Neuromuscular fatigue and recovery in elite female handball players. Scand J Med Sci Sports.

[16] Orchard J (2012). More research is needed into the effects on injury of substitute and interchange rules in team sports. Br J Sports Med.

[17] von Elm E, Altman DG, Egger M, Pocock SJ, Gøtzsche PC, Vandenbroucke JP, Initiative ftS (2007). The Strengthening the Reporting of Observational Studies in Epidemiology (STROBE) Statement: Guidelines for Reporting Observational Studies. Epidemiology.

[18] Spiegelman D, Hertzmark E (2005). Easy SAS calculations for risk or prevalence ratios and differences. Am J Epidemiol.

[19] Barros AJ, Hirakata VN (2003). Alternatives for logistic regression in cross-sectional studies: an empirical comparison of models that directly estimate the prevalence ratio. BMC Med Res Methodol.

[20] Brumback BA, Dailey AB, Brumback LC, Livingston MD, He Z (2010). Adjusting for confounding by cluster using generalized linear mixed models. Statistics & Probability Letters.

[21] Barnard J, Meng XL (1999). Applications of multiple imputation in medical studies: from AIDS to NHANES. Stat Methods Med Res.

[22] Vandenbroucke JP, Elm E, Altman DG, Gøtzsche PC, Mulrow CD, Pocock SJ, Poole C, Schlesselman JJ, Egger M (2007). Strengthening the Reporting of Observational Studies in Epidemiology (STROBE): Explanation and Elaboration. Ann Intern Med.

[23] Theisen D, Frisch A, Malisoux L, Urhausen A, Croisier JL, Seil R (2013). Injury risk is different in team and individual youth sport. J Sci Med Sport.

[24] Luke A, Lazaro RM, Bergeron MF, Keyser L, Benjamin H, Brenner J, d’Hemecourt P, Grady M, Philpott J, Smith A (2011). Sports-related injuries in youth athletes: is overscheduling a risk factor?. Clin J Sport Med.

[25] Dollman J, Norton K, Norton L (2005). Evidence for secular trends in children’s physical activity behaviour. Br J Sports Med.

[26] Martin M, Dollman J, Norton K, Robertson I (2005). A decrease in the association between the physical activity patterns of Australian parents and their children; 1985-1997. J Sci Med Sport.

[27] Japan Sports Association, SASAKAWA SPORTS FOUNDATION: Report: the change in the numbers of Junior Sports Clubs and its members (2002-2014). 2016. http://www.ssf.or.jp/Portals/0/resources/research/report/pdf/report_201610_all.pdf. Accessed 12 Oct 2016.

[28] Howie EK, McVeigh JA, Smith AJ, Straker LM (2016). Organized Sport Trajectories from Childhood to Adolescence and Health Associations. Med Sci Sports Exerc.

[29] Vella SA, Cliff DP, Magee CA, Okely AD (2014). Sports participation and parent-reported health-related quality of life in children: longitudinal associations. J Pediatr.

[30] Slater A, Tiggemann M (2010). “Uncool to do sport”: A focus group study of adolescent girls’ reasons for withdrawing from physical activity. Psychol Sport Exerc.

[31] Sato T, Ito T, Hirano T, Morita O, Kikuchi R, Endo N, Tanabe N (2011). Low back pain in childhood and adolescence: assessment of sports activities. Eur Spine J.

[32] Paananen MV, Taimela SP, Auvinen JP, Tammelin TH, Kantomaa MT, Ebeling HE, Taanila AM, Zitting PJ, Karppinen JI (2010). Risk factors for persistence of multiple musculoskeletal pains in adolescence: a 2-year follow-up study. Eur J Pain.

[33] Diepenmaat AC, van der Wal MF, de Vet HC, Hirasing RA (2006). Neck/shoulder, low back, and arm pain in relation to computer use, physical activity, stress, and depression among Dutch adolescents. Pediatrics.

[34] Sabiston CM, O’Loughlin E, Brunet J, Chaiton M, Low NC, Barnett T, O’Loughlin J (2013). Linking depression symptom trajectories in adolescence to physical activity and team sports participation in young adults. Prev Med.

