# Supplementary material for: Is being a regular player with fewer teammates associated with musculoskeletal pain in youth team sports? A cross-sectional study
Source: BMC Musculoskelet Disord. 2017 Mar 14;18:105. doi: 10.1186/s12891-017-1470-z (PMC5348770; doi:10.1186/s12891-017-1470-z)
Supplement: Additional file 1: — eQuestionnaire 1. Pain questionnaire for adolescents. (PDF 480 kb) [file 12891_2017_1470_MOESM1_ESM.pdf]

eQuestionnaire 1. Pain questionnaire for adolescents.

Questions about pain

For each part of your body, please answer about “pain” that you have felt recently.
If you do not have any pain, please circle “no pain”.

(Example) **0. (Location)** ＜ have pain ・ no pain → Proceed to 2. Upper limb ＞

(1) Is it because you were injured (e.g., fell, hit a person or an object)?

＜ Yes ・ No ＞

(2) How long have you had the pain?

＜ 1 year or longer ・ 3 months or less ・ 1 month or less ・ 1 week or less ＞

(3) How often do you feel the pain?

＜ everyday ・ several times a week ・ several times a month ・ several times a year ＞

The anatomical locations are shown in black.
↓

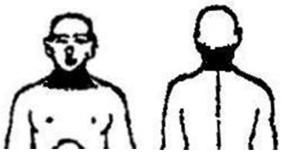

1. Neck ＜ have pain ・ no pain → Proceed to 2. Upper limb ＞

(1) Is it because you were injured (e.g., fell, hit a person or an object)?

＜ Yes ・ No ＞

(2) How long have you had the pain?

＜ 1 year or longer ・ ___months or less ・ 1 month or less ・ 1 week or less ＞

(3) How often do you feel the pain?

＜ everyday ・ several times a week ・ several times a month ・ several times a year ＞

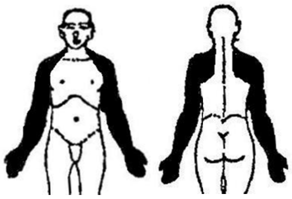

*If you have pain at two or more locations in your upper limbs, please answer only about one with the most severe (strongest) pain.

2. Upper limbs (shoulder, arms, hands) ＜ have pain ・ no pain → Proceed to 3.Chest ＞

(1) Is it because you were injured (e.g. fell, hit a person or an object)?

＜ Yes ・ No ＞

(2) How long have you had the pain?

＜ 1 year or longer ・ ___months or less ・ 1 month or less ・ 1 week or less ＞

(3) How often do you feel the pain?

＜ everyday ・ several times a week ・ several times a month ・ several times a year ＞

(4)At which location do you have the pain?

＜(e.g., shoulder, elbow, wrist, finger)____________________ ＞

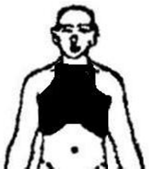

3. Chest ＜ have pain ・ no pain → Proceed to 4. Upper back ＞

(1) Is it because you were injured (e.g., fell, hit a person or an object)?

＜ Yes ・ No ＞

(2) How long have you had the pain?

＜ 1 year or longer ・ ___months or less ・ 1 month or less ・ 1 week or less ＞

(3) How often do you feel the pain?

＜ everyday ・ several times a week ・ several times a month ・ several times a year ＞

4. Upper back　＜ have pain ・ no pain → Proceed to 5. Lower back ＞

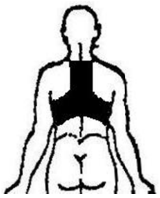

(1) Is it because you were injured (e.g., fell, hit a person or an object)?

＜ Yes ・ No ＞

(2) How long have you had the pain?

＜ 1 year or longer・___months or less・1 month or less・1 week or less ＞

(3) How often do you feel the pain?

＜ everyday ・ several times a week ・ several times a month ・ several times a year ＞

5. Lower back　＜ have pain ・ no pain → Proceed to 6. Buttock ＞

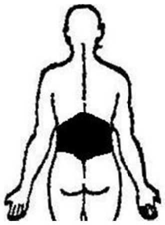

(1) Is it because you were injured (e.g., fell, hit a person or an object)?

＜ Yes ・ No ＞

(2) How long have you had the pain?

＜ 1 year or longer・___months or less・1 month or less・1 week or less ＞

(3) How often do you feel the pain?

＜ everyday ・ several times a week ・ several times a month ・ several times a year ＞

6. Buttocks　＜ have pain ・ no pain → Proceed to 7. Lower limb ＞

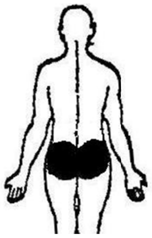

(1) Is it because you were injured (e.g., fell, hit a person or an object)?

＜ Yes ・ No ＞

(2) How long have you had the pain?

＜ 1 year or longer・___months or less・1 month or less・1 week or less ＞

(3) How often do you feel the pain?

＜ everyday ・ several times a week ・ several times a month ・ several times a year ＞

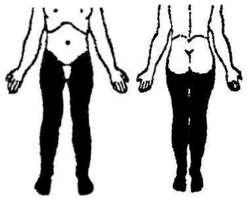

7. Lower limbs(thigh, hip, knee, foot, etc.)　＜ have pain ・ no pain ＞

(1) Is it because you were injured (e.g. fell, hit a person or an object)?

＜ Yes ・ No ＞

(2) How long have you had the pain?

＜ 1 year or longer・___months or less・1 month or less・1 week or less ＞

(3) How often do you feel the pain?

＜ everyday ・ several times a week ・ several times a month ・ several times a year ＞

(4)At which location do you have the pain?

＜(e.g., hip, knee, shin, ankle, instep, toe)__________________＞

*If you have pain at two or more locations in your lower limbs, please answer only about one with the most severe (strongest) pain.
